# The Effect of a Combined Mindfulness and Yoga Intervention on Soldier Mental Health in Basic Combat Training: A Cluster Randomized Controlled Trial

**DOI:** 10.1155/2023/6869543

**Published:** 2023-12-01

**Authors:** Thomas H. Nassif, Ian A. Gutierrez, Carl D. Smith, Amishi P. Jha, Amy B. Adler

**Affiliations:** ^1^Walter Reed Army Institute of Research, 503 Robert Grant Avenue, Silver Spring, MD 20910, USA; ^2^University of Miami, Coral Gables, FL 33124, USA

## Abstract

**Background:**

Depression, anxiety, and sleep problems are prevalent in high-stress occupations including military service. While effective therapies are available, scalable preventive mental health care interventions are needed. This study examined the impact of a combined mindfulness and yoga intervention on the mental health of soldiers in Basic Combat Training (BCT).

**Methods:**

U.S. Army soldiers (*N* = 1,896) were randomized by platoon to an intervention or training-as-usual condition. Soldiers in the intervention condition completed Mindfulness-Based Attention Training (MBAT), engaged in daily 15 min mindfulness practice, and participated in 30 minutes of hatha yoga 6 days per week. Surveys were administered at baseline (T1, prior to training), week 4 of BCT (T2), week 6 (T3), and week 9 (T4).

**Results:**

A significant time-by-condition interaction predicting positive screens for depression found that screens decreased at a faster rate from T1 to T4 in the intervention condition (-12.6%) compared to training-as-usual (-7.2%) (*b* = −0.18, SE = 0.07, *p* = 0.028). While positive anxiety screens decreased over time across conditions, the time-by-condition interaction found no significant differences in the rate of these decreases by condition (*b* = 0.09, SE = 0.09, *p* = 0.273). A significant time-by-condition interaction predicting positive screens for sleep problems found that sleep problems decreased in the intervention condition (-1.4%) but increased in training-as-usual (2.0%) (*b* = −0.68, SE = 0.16, *p* = 0.027).

**Conclusion:**

The mindfulness and yoga intervention was associated with a greater reduction in positive screens for depression and sleep problems among soldiers during high-stress training. Limitations include reliance on self-report and the inability to disaggregate the effects of mindfulness versus yoga. Mindfulness and yoga may enable personnel in high-stress occupations to sustain their mental health even in the context of significant psychological demands. This trial is registered with NCT05550610.

## 1. Introduction

Depression, anxiety, and sleep problems are prevalent in high-stress occupations including health care [1, 2], first responding [3, 4], and military service [5]. These mental health concerns are associated with missed work days and impaired workplace functioning [6]. Given the prevalence and implications of these mental health problems in high-stress occupations like military service, interventions are urgently needed.

While effective therapies are available in the military health system [7], the demands on health care resources [8, 9] signal the need for scalable, upstream interventions that can be implemented early in a soldier's career. To address this need for preventive mental health care strategies and early intervention in the military [10], the present study examined the impact of a combined mindfulness and yoga intervention on mental health among soldiers in Basic Combat Training (BCT).

The U.S. Army chose to evaluate a combined mindfulness and yoga intervention because they wanted to leverage the benefits of both interventions while taking into account funding constraints, timeline considerations, and platoon availability. These two interventions provide complementary skill sets that offer a potentially robust foundation for occupational stress tolerance and primary mental health prevention among service members. Consistent with this integration of the two interventions, some commonly researched mindfulness programs include postural yoga components [11, 12], and many postural yoga programs incorporate elements of mindfulness [13, 14].

Mindfulness is a mental mode involving focused attention to the present-moment without elaboration or emotional reactivity [12, 15]. Mindfulness training programs can enhance concentrative attention, cognitive monitoring, and openness to experience to help decrease mind-wandering and manage stress [16]. Ample evidence supports the effectiveness of mindfulness training for addressing mental health in clinical contexts [17, 18]. Mindfulness training has also been found to reduce anxiety and depression among personnel across a range of job sectors [19]. In addition, mindfulness training has been found to improve sleep quality and duration, alleviate insomnia symptoms, and mitigate cognitive processes that interfere with sleep, such as worry and rumination among civilians and veterans [20, 21].

Although some studies with military personnel have not found an impact of mindfulness training on anxiety and depression symptoms [22, 23], studies have found that greater frequency of mindfulness practice has led to improved emotional health among service members, such as positive mood [15] and emotion regulation [22]. Mindfulness intervention studies have operationalized formal mindfulness practice as a structured session in which participants follow a prerecorded mindfulness exercise. While formal practice is beneficial [24], it may be important to supplement training programs with embedded practices that are incorporated into work activities. Although little is known about the effects of embedding practice, this research gap is essential to address given the potential advantage of bringing mindfulness into work-related activities.

Another intervention that may be useful in supporting service member mental health is postural yoga. Postural yoga emphasizes physical postures and breathing techniques that have been used to support mental well-being [25]. Although yoga intervention studies predominantly employ physical postures as the core component of the study intervention, other practices (e.g., meditation [14]) are occasionally included as part of a multicomponent yoga intervention.

While there is evidence that postural yoga interventions improve depression symptoms in clinical samples [26], the effectiveness of yoga interventions for anxiety disorders and sleep problems is limited [13]. Still, yoga interventions have been found to decrease time to fall asleep and increase the number of hours slept [27]. With respect to the occupational context, yoga interventions in workplace settings are associated with improvements in anxiety symptoms and sleep quality, but not changes in depression [28].

These findings have been extended to high-stress work environments. For example, studies conducted in the Indian military have found that yoga was associated with decreases in anxiety symptoms and improvements in sleep quality [29, 30]; however, in these studies, yoga was provided during an intensive residential program that included other components (e.g., guided relaxation, prayer, chanting, and philosophy [29]). This larger, intensive program demonstrates the need to assess the feasibility of scaling yoga for a broader military context.

The present study reports on a cluster randomized controlled trial evaluating a combined mindfulness training and postural yoga intervention piloted by the U.S. Army during BCT. Incoming service members are less likely to have preconceived notions of current Army training and receive instructions in a highly structured environment. Thus, BCT was selected to enable the implementation of a novel training method that could help new soldiers manage occupational stress and prevent mental health problems. Randomization occurred at the platoon level to be consistent with military training delivered within the BCT context.

To our knowledge, this is the largest randomized trial to examine the effectiveness of mindfulness and yoga interventions on mental health outcomes in the military context. Given the prevalence of depression, anxiety, and sleep problems and the lack of scalable, preventive mental health care strategies in the military, findings from this study may inform primary mental health prevention among service members. In addition, this study addresses the literature gaps on mindfulness practices that are embedded into work activities. Such embedded practices can inform how formal training programs can be implemented into an occupational setting. Furthermore, there is limited research on the effects of a scalable yoga intervention conducted with military populations. More broadly, no previous research has examined the impact of mindfulness training and yoga among a large cohort of basic combat trainees.

Our primary aim was to determine the effect of a mindfulness and yoga intervention on mental health outcomes. We predicted that soldiers receiving the mindfulness and yoga intervention would be less likely to screen positive for depression, anxiety, and sleep problems over time compared to the training-as-usual condition. Our secondary aim was to determine the effectiveness of embedded mindfulness practice on these outcomes; we predicted that more frequent embedded mindfulness practice would lead to fewer positive screens. Although this study design did not allow for distinguishing between the unique effects of mindfulness training and yoga, the overall purpose of this study was to inform health promotion efforts regarding the potential benefits of implementing a combined mindfulness and yoga intervention during BCT and to guide the development of future intervention programming.

## 2. Materials and Methods

### 2.1. Participants

The study was designed as a cluster randomized trial. Participants were U.S. Army soldiers (*N* = 1,896) from two battalions who attended BCT between October 2020 and December 2020. BCT introduces enlisted soldiers to the Army by teaching them foundational soldier skills. The structured daily training schedule ranges from 12 to 15 hours per day for ten weeks. Soldiers live together in barracks and work together in platoons. During BCT, soldiers are required to become proficient in a series of tasks including hand-to-hand combat, rifle marksmanship, combat lifesaver skills, and land navigation. This training occurs in classrooms, ranges, and field environments.

In BCT, each battalion consisted of five companies, and each company consisted of four platoons. In the Basic Combat Training context, soldiers are arbitrarily assigned to platoons, companies, and battalions without regard to individual characteristics. Since the Army randomly assigns soldiers to platoons, we randomized platoons one and two to mindfulness training and yoga and platoons three and four to training-as-usual. This approach facilitated the military and research team in tracking a large number of units as part of implementing an evaluation in the real world. In all, 20 platoons were assigned to mindfulness and yoga (*n* = 813), and 20 platoons were assigned to training-as-usual (*n* = 771). Soldiers in both conditions participated in the evaluation throughout the ten weeks of BCT. Participant demographics reflected a typical BCT population.  Table 1 provides demographic details for the sample along with comparisons by condition.

### 2.2. Intervention

Mindfulness-Based Attention Training (MBAT) is an 8-hour manualized mindfulness intervention developed and contextualized for delivery to military personnel [16, 23, 31]. MBAT was delivered in a classroom setting or comparable low-distraction environment. The course consists of 2-hour sessions each week for 4 weeks, reflecting four themes: (1) breath awareness and focused attention skills, (2) body awareness without judgment, (3) open monitoring to observe sensory and mental experiences, and (4) interpersonal connection. Each session introduced a 15-minute mindfulness practice: (1) focused attention, (2) mindful body scan, (3) open monitoring, and (4) connection. Group mindfulness practice 6 days per week consisted of listening to a 15-minute audio recording. Each session also presented an exercise for embedding mindfulness practice during the day.

MBAT was delivered by masters- or doctoral-level performance experts (*n* = 10) who had an average of 4.40 years (SD = 3.62) of experience working within the military and little to no prior experience with mindfulness. Instructors attended 26 hours of virtual training sessions, participated in five hours of small group activities over the course of 12 weeks, and were assigned 15 minutes of daily mindfulness practice. Two instructors were assigned to each intervention platoon; each platoon was divided in half to ensure a smaller instructor-to-soldier ratio.

The postural yoga program consisted of hatha yoga postures that replaced exercises normally included at the beginning (preparatory drills) and end (recovery drills) of daily Physical Readiness Training (PRT). Yoga postures were designed to engage major muscle groups during the preparatory drills (e.g., sun salutations, crescent lunge, and eagle pose) and to release tension during the recovery drills (e.g., gate pose, reverse plank, and bridge pose). Online resources provide additional information about these yoga postures [32, 33].

Yoga instructors (*n* = 10) had at least two years of teaching experience and were registered with Yoga Alliance at the Registered Yoga Teacher (RYT) 200-hour level or higher. One yoga instructor was assigned to each intervention platoon. Yoga instructors completed 8 hours of virtual training sessions, which focused on teaching a standardized sequence of yoga postures aligned with the physical demands of BCT, linking movement to the breath, stabilizing core muscles, building lower and upper body strength, and reducing tension in the low back and hips.

### 2.3. Procedure

Soldiers in the intervention condition completed the MBAT course during the first four weeks of BCT, engaged in daily 15 min mindfulness practice with their platoon, were instructed on embedding individual mindfulness practice into the duty day, and participated in 30 minutes of hatha yoga 6 days per week. While platoons in the intervention condition were participating in intervention activities, platoons assigned to training-as-usual either were instructed to use the time to review BCT material or engaged in standard preparatory and recovery drills. The standard preparatory drills consisted of exercises (e.g., rear lunge, high jumper, and squat bender) designed to increase muscular endurance, reduce injury risk, and improve overall fitness. The standard recovery drills consisted of stretches (e.g., overhead arm pull, extend and flex, and thigh stretch) to develop flexibility across major muscle groups including the shoulders, hip flexors, and lower back.

**Figure 1:**
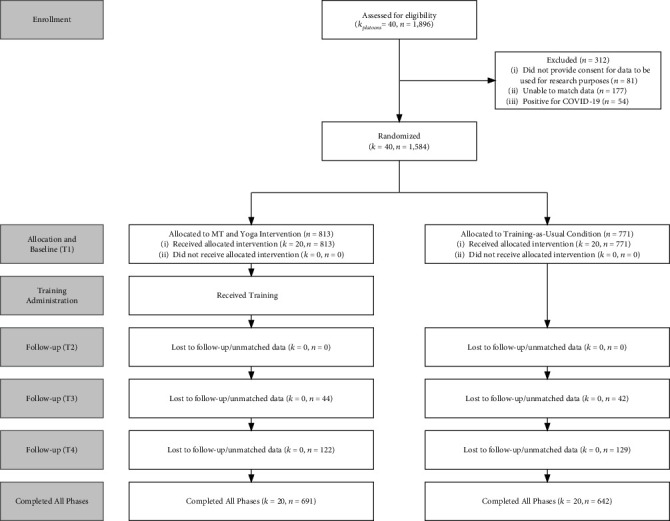
CONSORT diagram.

All soldiers were briefed prior to study enrollment, and soldiers who provided informed consent for research purposes did so prior to completing the baseline survey. Soldiers were first assigned to a residential bay upon arrival at BCT; after three weeks, bays were then allocated to larger platoons. Bays were randomized to either an intervention or training-as-usual platoon. Participant exclusion, randomization, and attrition numbers are presented in the CONSORT diagram in Figure 1. Of the 1,896 soldiers in the evaluation, data from 312 (16.5%) were excluded from analysis: 81 did not consent to have their survey responses used for research purposes, 177 could not be matched to condition assignment by their unique identifier, and 54 were assigned to a bay of COVID-19 positive soldiers.

There were four surveys: baseline (T1, prior to training), week 4 of BCT (T2), week 6 (T3), and week 9 (T4). Soldiers in the intervention condition also completed a course evaluation at T4. Completion of surveys was voluntary. Soldiers were instructed that there were no penalties for choosing not to participate and they would not be compensated for participation. Soldiers were informed that their responses were private and confidential unless they indicated intent to harm self or others. To minimize coercion or undue influence, drill sergeants and other unit leadership were not present during the consent process. Study procedures were conducted in accordance with Army Regulation 70-25 [34]. As an exempt study, surveys and procedures were approved by the Human Subjects Protection Branch at the Walter Reed Army Institute of Research.

### 2.4. Inclusion Criteria

Soldiers attending BCT at one military installation in the Southeastern United States were eligible for inclusion.

### 2.5. Exclusion Criteria

Soldiers who tested positive for COVID-19 during routine BCT medical screening were excluded from analysis.

## 3. Measures

### 3.1. Demographics

At T1, soldiers were asked demographic questions. They were asked their current age with response options across four categories (18-19, 20-24, 25-29, and 30+). In terms of gender, they were asked if they were male or female. They were also asked their highest level of civilian education with response options across four categories (high school or equivalent, some college or associate degree, bachelor's degree, and graduate degree).

### 3.2. Mindfulness and Yoga History

At T1, soldiers were asked how many times they had practiced mindfulness or yoga before BCT using responses ranging from 0 (*never*) to 3 (*many times*). The mindfulness item and the yoga item were dichotomized into “no prior experience” and “prior experience,” with responses greater than “never” being coded as “prior experience.”

### 3.3. Depression

Depression was assessed with the two-item Patient Health Questionnaire (PHQ-2) [35] rated from 0 (*not at all*) to 3 (*nearly every day*). The two items were summed, and scores of 3 or greater were coded as positive screens. Scores were not tabulated if data were missing for either item. The PHQ-2 has been shown to be a reliable and valid measure in previous studies [35–37].

### 3.4. Anxiety

Anxiety was measured with the two-item Generalized Anxiety Disorder (GAD-2) scale [38] rated from 0 (*not at all*) to 3 (*nearly every day*). The two items were summed, and scores of 3 or greater were coded as positive screens. Scores were not tabulated if data were missing for either item. The GAD-2 has been shown to be a reliable and valid measure in previous studies [36, 39].

### 3.5. Sleep Problems

Sleep problems were measured using four items from the Insomnia Severity Index [40]; this shortened form has been used in other military studies [41]. Soldiers rated their sleep over the past two weeks with respect to their difficulty falling asleep and difficulty staying asleep from 1 (*none*) to 5 (*very severe*), their satisfaction with their current sleep pattern from 1 (*very dissatisfied*) to 5 (*very satisfied*), and the extent to which their sleep interfered with their daily functioning from 1 (*not at all/no sleep problem*) to 5 (*very much*). Positive screens were based on scoring “at risk” on at least 3 items [41]. Scores were not tabulated if data were missing for any item.

### 3.6. Individual Mindfulness Practice during BCT

On the course evaluation survey, soldiers were asked, “On average, how many days a week did you practice MBAT on your own?” Response options were “0 days,” “1-2 days,” “3-4 days,” “5-6 days,” and “7 days.” Soldiers who reported practicing 1-2 days per week or less were classified as “low practice,” while those who reported practicing 3-4 days per week or more were classified as “high practice.”

## 4. Statistical Analysis

Frequencies were calculated for sample demographics, and chi-square tests were conducted to test demographic differences between conditions to identify potential covariates for subsequent models.

The percentage of soldiers screening positive for depression, anxiety, and sleep problems by condition was computed for each time point. We employed binomial generalized linear mixed models (GLMMs) to predict the likelihood of positive screens by time and condition while accounting for potential clustering effects of platoon membership. GLMMs were estimated using restricted maximum likelihood estimation with bound optimization by quadratic approximation. Each GLMM was specified as a three-level longitudinal model, with time nested within soldier and soldiers nested within platoons. Random intercepts and uncorrelated random slopes were estimated for all models. Interclass correlation coefficients (ICCs) were computed to assess the proportion of variance explained by the random effects of soldier and platoon.

For each outcome, positive mental health screens were first modeled as a function of the interaction between time and condition, with training-as-usual defined as the referent group (hereafter referred to as “time-by-condition” GLMMs). Subsequently, to examine how the frequency of individual embedded mindfulness practice impacted positive mental health screens, we then ran another set of GLMM modeling positive screens as a function of an interaction between time and condition, with the mindfulness and yoga condition divided into the low practice and high practice groups (hereafter referred as “time-by-practice” GLMMs). Across all six GLMMs—three time-by-condition models and three time-by-practice models—training-as-usual was treated as the reference group.

Across all conditions and time points, items assessing depression, anxiety, and sleep problems had no more than 3.6% missing data. Missing data for positive screens were 4.1% for depression, 4.1% for anxiety, and 2.7% for sleep problems. The individual mindfulness practice item had 5.2% missing data. For chi-square analyses conducted on demographic data, listwise deletion was used to remove respondents who did not provide information. For the time-by-condition and time-by-practice models, missing data were handled through intent-to-treat analyses through the use of GLMMs [42].

We conducted an a priori multilevel power analysis using Optimal Design software [43]. Analyses found that power exceeded 0.8 for an estimated effect size of *d* = 0.18 with cluster ICCs not exceeding 0.01 (*k* = 40 platoons and, approximately, *n* = 40 soldiers per platoon). This result indicated that our design would be adequate to identify small to medium effects.

All analyses were conducted in R v.4.1.0 [44]. GLMMs were estimated using the “lme4” package [45].

## 5. Results

No significant condition-wise differences were observed with respect to demographics and mindfulness and yoga history (Table 1). Thus, these variables were excluded from further analyses. Among soldiers in the intervention condition who indicated their frequency of individual embedded mindfulness practice (83.0%, *n* = 677), 37.0% (*n* = 249) reported high individual practice.

### 5.1. Depression

ICCs for depression were 0.53 for the random effect of soldier and less than 0.01 for the random effect of platoon, indicating that depression outcomes shared intraindividual variability across time but evidenced little clustering by platoon. The time-by-condition model found a significant main effect of time, such that positive depression screens decreased over time across all soldiers (Table 2). However, the model also revealed a significant interaction between time and condition, such that the proportion of positive screens among soldiers in the mindfulness and yoga condition decreased at a significantly faster rate than did the proportion of positive screens among soldiers in the training-as-usual condition: from T1 to T4, positive screens fell 12.6% in the mindfulness and yoga condition, whereas positive screens fell 7.2% in the training-as-usual condition (see Figure S1A).

**Figure 2:**
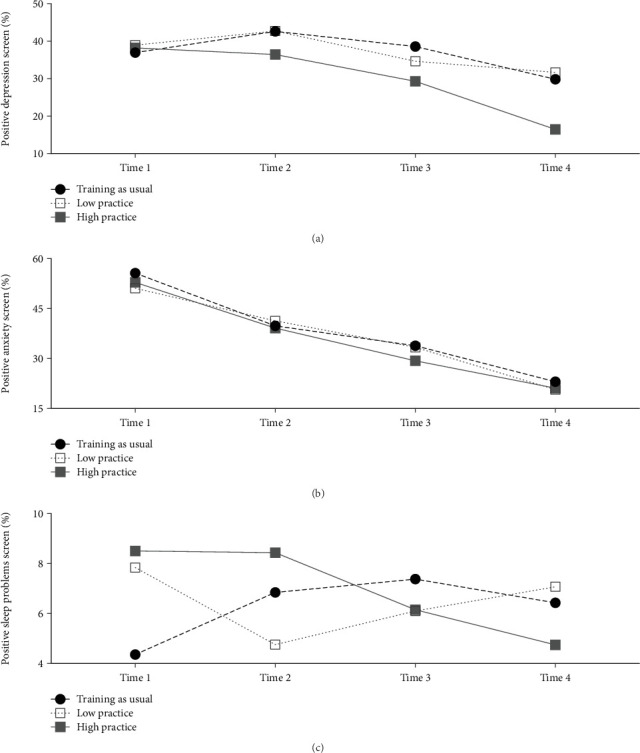
Percent of trainees' meeting screening criteria for depression, anxiety, and sleep problems over time by interaction of embedded practice frequency and condition.

The time-by-practice model revealed a significant time-by-practice interaction, such that the proportion of soldiers screening positive in the high practice group decreased at a faster rate compared to soldiers in training-as-usual (Table 2). There was no significant difference in positive screens over time between the low practice group and training-as-usual. From T1 to T4, positive screens in the high practice group fell 21.8%, whereas positive screens in the low practice group fell 7.3% (Figure 2(a); confidence interval bands are provided in Figure S2A).

### 5.2. Anxiety

ICCs for anxiety were 0.47 for the random effect of soldier and less than 0.01 for the random effect of platoon. The time-by-condition model identified a significant main effect of time on positive screens, such that anxiety decreased across the entire study sample (Table 2). Neither a significant effect of condition nor a significant time-by-condition was observed (see Figure S1B).

The time-by-practice model also did not yield significant group- or condition-wise differences. The proportion of soldiers screening positive among the high practice group, low practice group, and training-as-usual condition decreased 31.7%, 30.5%, and 32.6% from T1 to T4, respectively (Figure 2(b); confidence interval bands are provided in Figure S2B).

### 5.3. Sleep Problems

ICCs for sleep problems were 0.91 for the random effect of soldier and less than 0.01 for the random effect of platoon. The time-by-condition model revealed a significant main effect of time, indicating a significant decrease in positive screens across conditions. We also observed a significant main effect of condition, highlighting the overall higher level of sleep problems among the intervention condition, which is most evident at T1 where sleep problems were 3.4% higher in the intervention condition compared to training-as-usual. A significant time-by-condition interaction reflected differences in condition-wise slopes, such that sleep problems in the intervention condition decreased 1.4% from T1 to T4, whereas sleep problems in training-as-usual increased 2.0% (see Figure S1C).

Figure 2(c) depicts the time-by-practice model for sleep problems, demonstrating a significant interaction such that the proportion of soldiers with sleep problems in the high practice group decreased, whereas sleep problems among soldiers in training-as-usual increased (confidence interval bands are provided in Figure S2C). This model found no significant difference in sleep problems over time between the low practice group and training-as-usual, indicating that the significant interaction observed in the time-by-condition model is better understood as a being driven by the high practice group. From T1 to T4, sleep problems in the high practice group fell 3.8%, whereas positive screens in the low practice group fell 0.7%.

## 6. Discussion

Soldiers assigned to a combined mindfulness and yoga training during BCT were less likely to have positive screens for depression or sleep problems over time compared to those receiving training-as-usual. Despite baseline differences in sleep problems, analyses examining a time-by-condition interaction, which accounts for baseline differences, revealed a significant effect for the intervention condition. Findings were consistent with prior research demonstrating the salutary effects of mindfulness training on depression [17, 18, 46, 47] and insomnia symptoms [20, 21] and of postural yoga on depression symptoms [26] and sleep quality [27].

We also found that positive screens for depression and sleep problems decreased more rapidly over time among soldiers who engaged in individual embedded mindfulness practice at least three days per week. Given that this study employed a formalized group practice intervention in line with previous research [22, 23], these findings add to existing knowledge by demonstrating that mindfulness training can be strengthened through frequent individual embedded practice in the workplace. Study results suggest that it may be useful to integrate mindfulness practice into smaller moments during the day beyond a single 15-minute session. It may be that when soldiers chose to embed mindfulness training, they were also identifying what they could control in a context in which many demands are outside of their control. Learning how to distinguish between what one can and cannot control may be particularly useful in a high-stress environment like the military. Importantly, future research should examine the benefits of integrating yoga postures throughout the day; unfortunately, this pattern of behavior was not assessed in the present study.

A number of mechanisms may explain the more rapid decrease in positive screens for depression and sleep problems in the intervention group. Focusing attention on one's present-moment experience from a nonjudgmental perspective could help to decrease self-criticism and improve mood [48]. Mindfulness training may also mitigate cognitive processes that interfere with sleep such as rumination [20, 21]. Additionally, learning new skills through a mindfulness training and postural yoga program could support mental health through increased self-esteem and a reduction in the frequency or intensity of negative thoughts [49].

While positive screens for anxiety decreased over time for all participants, there were no significant differences between conditions. The absence of a significant time-by-condition or time-by-practice finding for anxiety in the present study aligns with previous research on postural yoga [13]; however, findings are not consistent with previous research showing that mindfulness training reduced anxiety symptoms in some occupational settings [19]. It may be that the reduction in stress soldiers typically experience over the course of BCT [50, 51] accounted for the absence of differences by training condition. Interestingly, anxiety was endorsed by the majority of study participants and the most commonly reported mental health concern. For most soldiers, anxiety may be fueled by the series of different and unique demands encountered during BCT. Stress theory posits that novelty is a primary driver of anxiety [52]. Thus, it may be that soldiers acclimated to the environment over time, and this adaptation led to reduced anxiety rather than differences associated with training condition.

### 6.1. Limitations

A few limitations to the study merit discussion. First, we relied on self-report for assessing mental health. Second, frequency of individual embedded yoga practice was not tracked, so the impact of embedded yoga practice outside of PRT is unknown. Third, all soldiers assigned to the intervention condition received both mindfulness training and postural yoga. Given that many mindfulness programs include postural yoga components [11, 53] and postural yoga programs often incorporate elements of mindfulness [13, 14], mindfulness and yoga might separately contribute to mental health, have a synergistic effect, or have differential impacts on various outcomes. Future research should differentiate between the impacts of mindfulness training and postural yoga on mental health.

## 7. Conclusions

To our knowledge, the present study represents the largest systematic effort to explore the impact of mindfulness and yoga on mental health among personnel in a high-risk occupation. Our findings provide evidence for the positive impact of a combined mindfulness and yoga intervention on depression and sleep problems among soldiers during high-stress training and highlight the added benefits of embedding mindfulness practice into everyday life. Positive findings are also noteworthy given that the mindfulness trainers had no or limited previous background in mindfulness, suggesting that this approach may be scalable for large organizations like the military. Future research should explore how the use of embedded practices can be supported in the workplace and how other outcomes related to personnel well-being might be impacted. Given that individual embedded practices were conducted in combination with formal group practice, future research should examine the relative unique benefit of each. Mindfulness and yoga may enable personnel in high-stress occupations, such as health care and first responders, to sustain their mental health even in the context of significant psychological demands.

## Figures and Tables

**Table 1:** Demographics by condition.

Variable	Total	Condition		Test statistic	*p*
		Mindfulness and yoga	Training-as-usual		
	*N* (percent)	*N* (percent)	*N* (percent)		
*N*	1584	813	771		
Age				*χ* ^2^(3) = 0.32	0.956
18-19	664 (42.8%)	341 (43.2%)	323 (42.4%)		
20-24	525 (33.8%)	262 (33.2%)	263 (34.5%)		
25-29	221 (14.2%)	114 (14.4%)	107 (14.0%)		
30+	142 (9.1%)	73 (9.2%)	69 (9.1%)		
Education				*χ* ^2^(3) = 6.42	0.093
HS/GED	908 (57.6%)	462 (57.1%)	446 (58.1%)		
Some college or associates	449 (28.5%)	220 (27.2%)	229 (29.8%)		
Bachelors	175 (11.1%)	105 (13.0%)	70 (9.1%)		
Graduate	45 (2.9%)	22 (2.7%)	23 (3.0%)		
Gender				*χ* ^2^(1) ≤ 0.01	>0.999
Male	1142 (72.2%)	586 (72.3%)	556 (72.2%)		
Female	439 (27.8%)	225 (27.7%)	214 (27.8%)		
Past yoga practice	777 (49.8%)	409 (50.9%)	368 (48.6%)	*χ* ^2^(1) = 0.75	0.387
Past mindfulness practice	749 (48.1%)	383 (47.8%)	366 (48.4%)	*χ* ^2^(1) = 0.04	0.835
Baseline mental health					
Positive depression screens	589 (37.9%)	310 (38.8%)	279 (37.0%)	*χ* ^2^(1) = 0.46	0.498
Positive anxiety screens	825 (53.2%)	405 (50.9%)	420 (55.6%)	*χ* ^2^(1) = 3.32	0.068
Positive sleep problem screens	94 (6.0%)	61 (7.6%)	33 (4.4%)	*χ* ^2^(1) = 6.74	0.009⁣^∗∗^

Note. Percentages reported as a total of valid responses. ⁣^∗^*p* < 0.05, ⁣^∗∗^*p* < 0.01, and ⁣^∗∗∗^*p* < 0.001.

**Table 2:** Generalized linear mixed effect models predicting mental health screens from time-by-condition and time-by-practice interactions.

	Depression				Anxiety				Sleep problems			
	*b*	SE	*p*	OR [95% CI]	*b*	SE	*p*	OR [95% CI]	*b*	SE	*p*	OR [95% CI]
Time-by-condition models												
Fixed effects												
Intercept	-0.32⁣^∗^	0.11	0.043	0.73 [0.54, 0.99]	1.27⁣^∗∗∗^	0.57	<0.001	3.56 [2.60, 4.88]	-7.27⁣^∗∗∗^	0.00	<0.001	0.00 [0.00, 0.00]
Time	-0.26⁣^∗∗^	0.06	0.001	0.77 [0.67, 0.89]	-0.92⁣^∗∗∗^	0.03	<0.001	0.40 [0.34, 0.46]	-0.78⁣^∗∗^	0.11	0.001	0.46 [0.29, 0.74]
Condition^1^	0.28	0.25	0.145	1.32 [0.91, 1.92]	-0.29	0.15	0.155	0.75 [0.50, 1.12]	1.44⁣^∗^	2.60	0.018	4.24 [1.28, 14.08]
Time × condition^1^	-0.18⁣^∗^	0.07	0.028	0.83 [0.71, 0.98]	0.09	0.09	0.273	1.10 [0.93, 1.29]	-0.68⁣^∗^	0.16	0.027	0.51 [0.28, 0.93]
Random effects	Variance				Variance				Variance			
Soldier-intercept	1.62				1.92				6.58			
Soldier-slope	0.54				0.43				2.81			
Platoon-intercept	<0.01				0.00				<0.01			
Platoon-slope	0.10				0.09				<0.01			
Time-by-practice models	*b*	SE	*p*	OR [95% CI]	*b*	SE	*p*	*p*	*b*	SE	*p*	OR [95% CI]
Fixed effects												
Intercept	-0.36⁣^∗^	0.11	0.021	0.70 [0.52, 0.95]	1.26⁣^∗∗∗^	0.57	<0.001	3.53 [2.58, 4.84]	-7.35⁣^∗∗∗^	0.00	<0.001	0.00 [0.00, 0.00]
Time	-0.24⁣^∗∗^	0.05	0.001	0.79 [0.69, 0.90]	-0.92⁣^∗∗∗^	0.03	<0.001	0.40 [0.34, 0.47]	-0.75⁣^∗∗^	0.11	0.002	0.47 [0.29, 0.76]
Low practice	0.12	0.26	0.592	1.13 [0.72, 1.78]	-0.26	0.19	0.275	0.77 [0.48, 1.23]	1.32	2.74	0.071	3.74 [0.89, 15.68]
High practice	0.55	0.49	0.053	1.73 [0.99, 3.02]	-0.15	0.25	0.598	0.86 [0.48, 1.52]	2.31⁣^∗∗^	8.29	0.005	10.08 [2.01, 50.49]
Time × low practice^1^	-0.07	0.09	0.441	0.93 [0.78, 1.12]	0.08	0.10	0.382	1.09 [0.90, 1.31]	-0.65	0.20	0.083	0.52 [0.25, 1.09]
Time × high practice^1^	-0.45⁣^∗∗∗^	0.07	<0.001	0.64 [0.51, 0.80]	-0.01	0.11	0.931	0.99 [0.79, 1.24]	-1.21⁣^∗^	0.15	0.013	0.30 [0.12, 0.77]
Random effects	Variance				Variance				Variance			
Soldier-intercept	1.72				1.90				6.70			
Soldier-slope	<0.01				<0.01				<0.01			
Platoon-intercept	0.47				0.43				2.79			
Platoon-slope	0.09				0.10				<0.01			

Note. ^1^Training-as-usual used as the referent group. ⁣^∗^*p* < 0.05, ⁣^∗∗^*p* < 0.01, and ⁣^∗∗∗^*p* < 0.001.

## Data Availability

The data that support the findings of this study are not currently publicly available due institutional regulations protecting service member survey responses but are available from the corresponding author on reasonable request (may require data use agreements to be developed).

## Supplementary Materials

Supplementary Materials Figure S1 shows percentages and confidence interval bands for positive screens for depression, anxiety, and sleep problems over time-by-condition. Figure S2 depicts percentages and confidence interval bands for positive screens for depression, anxiety, and sleep problems over time by interaction of embedded practice frequency and condition. Confidence interval bands in each figure were computed using Wilson's confidence interval for binomial proportions.
