# Supplementary figures and images for: The Effect of a Combined Mindfulness and Yoga Intervention on Soldier Mental Health in Basic Combat Training: A Cluster Randomized Controlled Trial
Source: Depress Anxiety. 2023 Dec 1;2023:6869543. doi: 10.1155/2023/6869543 (PMC11921832; doi:10.1155/2023/6869543)

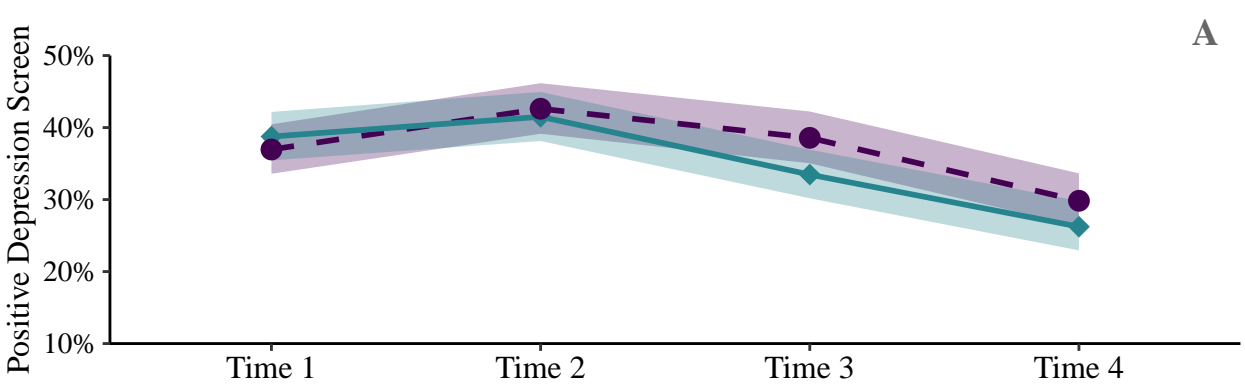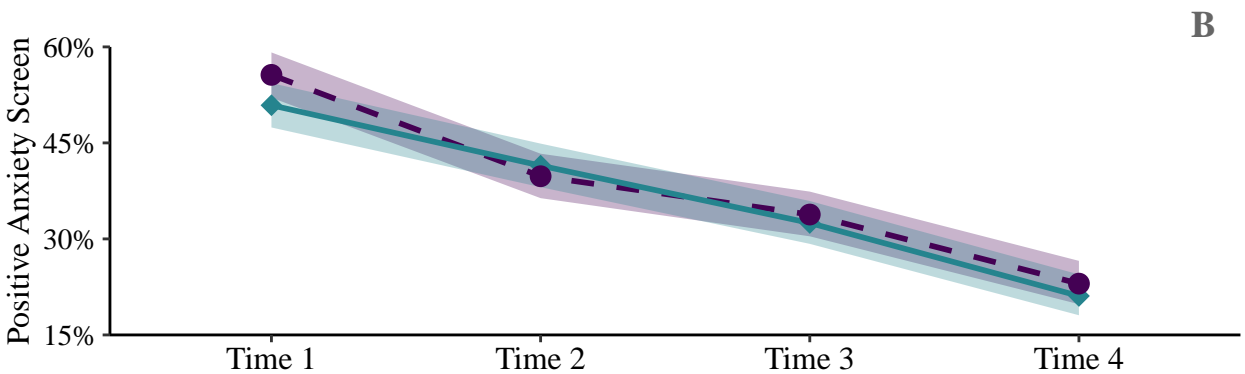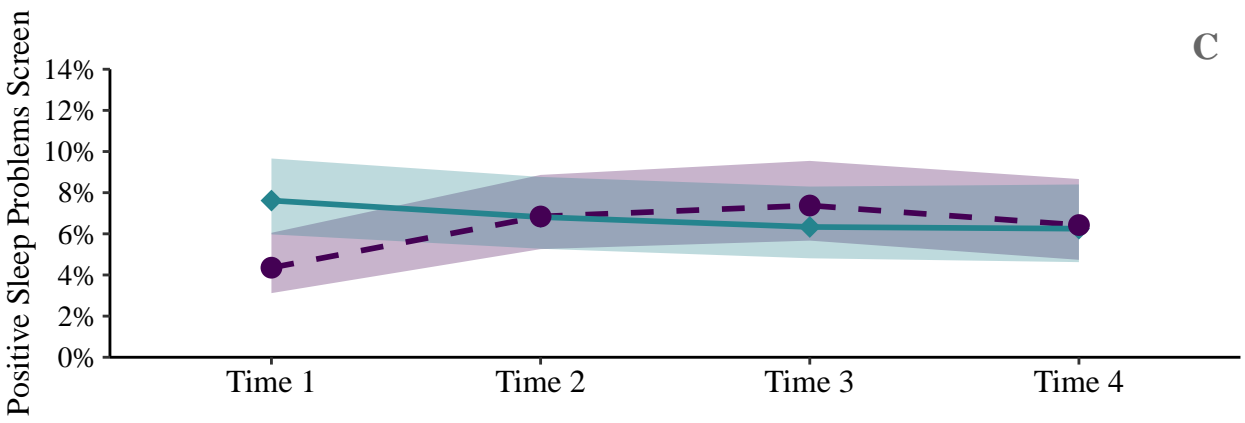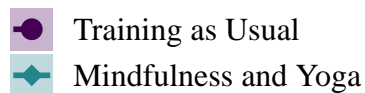

Supplement: Supplementary Materials — Figure S1 shows percentages and confidence interval bands for positive screens for depression, anxiety, and sleep problems over time-by-condition. Figure S2 depicts percentages and confidence interval bands for positive screens for depression, anxiety, and sleep problems over time by interaction of embedded practice frequency and condition. Confidence interval bands in each figure were computed using Wilson's confidence interval for binomial proportions. [file 6869543.f1.zip › Figure S1.pdf]

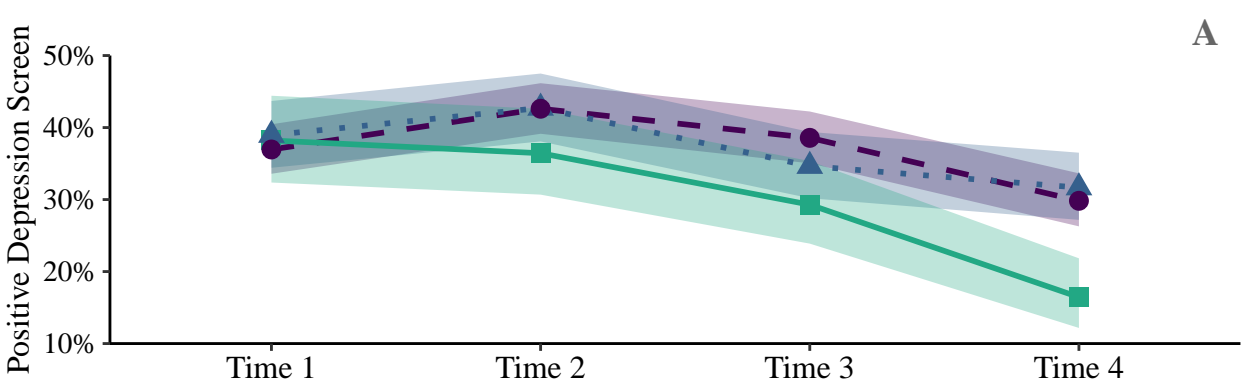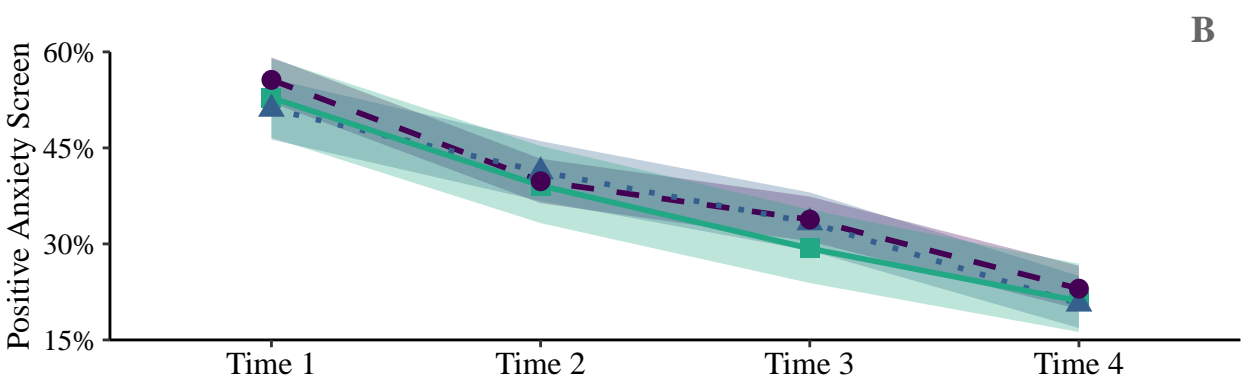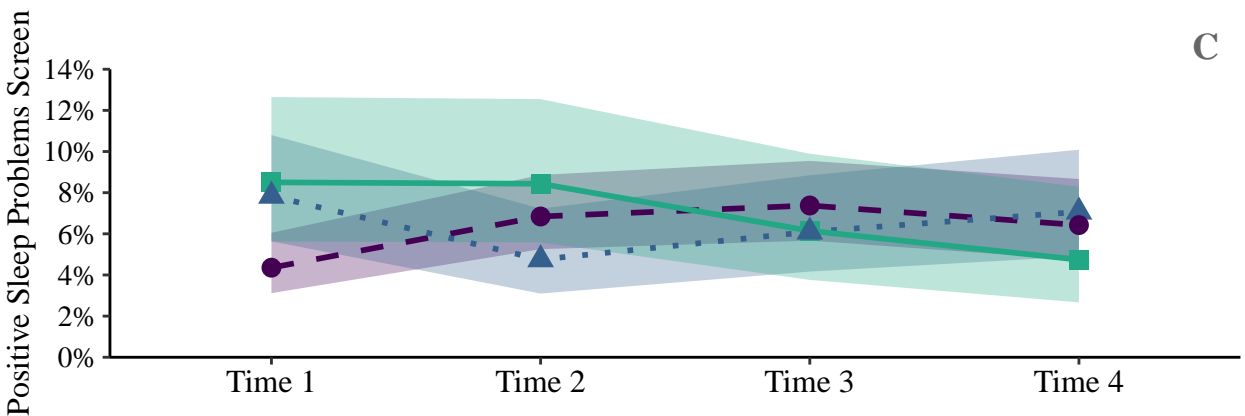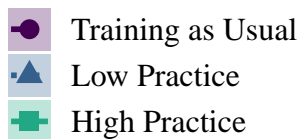

Supplement: Supplementary Materials — Figure S1 shows percentages and confidence interval bands for positive screens for depression, anxiety, and sleep problems over time-by-condition. Figure S2 depicts percentages and confidence interval bands for positive screens for depression, anxiety, and sleep problems over time by interaction of embedded practice frequency and condition. Confidence interval bands in each figure were computed using Wilson's confidence interval for binomial proportions. [file 6869543.f1.zip › Figure S2.pdf]
